# Avian influenza risk mapping in India using machine learning

**DOI:** 10.3389/fvets.2026.1842591

**Published:** 2026-08-05

**Authors:** Satish S. Gaikwad, T. R. Arun, Basavaraj Shrinivasa, Pragya Yadav, Babasaheb Tandale

**Affiliations:** 1 National Institute of One Health, Nagpur, India; 2 ICMR-National Institute of Virology, Pune, India

**Keywords:** avian influenza, India, machine learning, prediction uncertainty, spatial cross-validation, spatial epidemiology, spatial risk mapping

## Abstract

Highly pathogenic avian influenza (HPAI) remains a recurrent threat to poultry production and One-Health surveillance in India. We developed a national relative spatial risk map for India using curated outbreak records spanning January 2006 to April 2024 (predominantly HPAI H5N1 and H5N8), buffered pseudo-absence sampling, H3 resolution-7 hexagons, and 94 environmental, livestock, land-cover, and anthropogenic predictors. Under 5-fold spatial block cross-validation, eight base classifiers were trained and all performed above chance (AUC > 0.76). The Gaussian-process stacked meta-learner achieved the highest AUC (0.853), but the improvement over the strongest individual base learner, Random Forest (AUC 0.851; Brier 0.155; ECE 0.079), was small and statistically non-significant. Its principal added value was a companion uncertainty layer, the GPR posterior standard deviation, which showed internal consistency with ensemble disagreement across base models (*r* = 0.74, *p* < 0.001). Feature attribution ranked human population density, extensive chicken density, June precipitation, and seasonal humidity variables among the predictors most associated with model outputs, with interpretation constrained by passive-surveillance bias and multicollinearity. The resulting relative spatial risk surface, prediction-uncertainty surface, and subdistrict risk-uncertainty classification layers identify eastern, northeastern, coastal, and selected southern regions as priorities for targeted surveillance and prospective validation.

## 1 Introduction

Avian influenza (AI) viruses are maintained in wild aquatic birds and can spill over into domestic poultry, where some strains evolve into highly pathogenic forms. Highly pathogenic avian influenza (HPAI) subtypes such as H5N1, H5N8, and H7N9 cause substantial losses in poultry due to high mortality, culling, and trade restrictions (1). In addition, certain HPAI viruses can infect humans and are associated with severe disease, keeping avian influenza a continuing public health concern with pandemic potential (2, 3).

Machine-learning risk mapping for HPAI in India has been demonstrated at national scale using administrative-unit and environmental predictors (4), providing a foundation for higher-resolution, uncertainty-aware approaches. India has a rapidly intensifying poultry industry coexisting alongside traditional backyard systems, dense human populations often living in close proximity to livestock, and a position along major migratory bird flyways. The country has experienced recurrent HPAI outbreaks (5–7). The interface between wild birds, domestic poultry, environmental factors influencing virus survival and transmission, live bird market networks, and human activities creates a risk environment that requires tools for assessment and management. In response to this risk environment, pandemic preparedness has been identified as a national priority in India. Risk maps support this effort by enabling efficient targeting of surveillance activities and deployment of control measures (8–15).

Building on these methodological capabilities, we developed a national HPAI relative spatial risk map for India using H3 resolution-7 hexagons, curated outbreak records spanning 2006 to 2024, environmental and anthropogenic predictors, spatial block cross-validation, and an uncertainty-aware stacked modeling framework. The objectives were to evaluate predictive discrimination under spatial cross-validation, to quantify spatial uncertainty, to identify predictor groups most associated with model predictions, and to generate an operational map for surveillance prioritization. Following established infectious-disease risk-mapping approaches (4, 8–15), spatial risk mapping was used to estimate geographic variation in reported avian influenza occurrence under observed environmental, livestock, land-cover, and anthropogenic conditions. Because the model was trained using reported presence records and sampled pseudo-absence/background locations rather than verified true absences, the 0–1 model outputs should be interpreted as relative spatial risk or suitability scores for surveillance prioritization, not as absolute probabilities of avian influenza occurrence.

## 2 Materials and methods

### 2.1 Study area and geospatial framework

The study focused on the Republic of India. A standardized geospatial framework was established using the H3 global hexagonal hierarchical spatial index system at resolution 7 (~5.16 km^2^ hexagon area) covering the entire country (115,781 hexagons). India contains heterogeneous poultry production systems, including intensive commercial operations and extensive backyard flocks, often located near dense human settlements and wetland-agricultural interfaces (16). The country lies along migratory-bird flyways, including the Central Asian Flyway, creating contact opportunities among wild birds, domestic poultry, and human-managed environments. These ecological and production gradients motivated inclusion of poultry density, human population density, hydrological, land-cover, and seasonal climate predictors. Projections appropriate for India (UTM Zones 43 N/44 N/45 N) were used for all distance and area calculations.

### 2.2 Data sources and preparation

Confirmed avian influenza outbreak records in poultry, predominantly HPAI H5, were compiled from the World Organization for Animal Health (WOAH) WAHIS portal and FAO EMPRES-i. The source dataset contained 458 India records spanning January 2006 to April 2024, of which 456 were confirmed avian influenza outbreak records. By subtype, the confirmed records comprised H5N1 HPAI (393), H5N8 HPAI (55), and unspecified HPAI (8). After removing entries with missing or imprecise coordinates, 73 georeferenced outbreak records were retained within the study area. These outbreak records were then mapped to the H3 resolution-7 grid, corresponding to 56 unique presence hexagons. Published literature (6, 7) contributed an additional 15 unique H3 resolution-7 presence hexagons after deduplication, bringing the final presence set to 71 unique hexagons (56 from WOAH/EMPRES-i georeferencing + 15 from published literature after deduplication; see Supplementary Table S2 for a record-level accounting) (Table 1).

**Table 1:** List of predictor variables and their sources.

Variable class	Feature name	Primary data source
Anthropogenic	Human Population Density (2020; persons/km^2^)	WorldPop
	Urban and Built-up Land Cover (fraction 0–1)	ESA WorldCover
	Agricultural Expansion Areas (fraction 0–1)	Global Land Analysis and Discovery (GLAD)
Livestock	Domestic Duck Density (2015; heads/km^2^)	FAO GLW v4
	Intensive/Extensive Chicken Population Density (2015; heads/km^2^)	FAO GLW v4
	Gridded Livestock Density (Buffalo, Pigs, Poultry; heads/km^2^)	FAO GLW v4
Surface temperature	Day and Night Land Surface Temperature (LST; °C)	MODIS (MOD11A2)
	LST Harmonic Mean (Annual; °C)	MODIS (MOD11A2)
	LST Harmonic Variance (Annual, Semi-annual, Tri-annual; °C^2^)	MODIS (MOD11A2)
	LST Harmonic Amplitude (Annual, Semi-annual, Tri-annual; °C)	MODIS (MOD11A2)
Vegetation indices	Normalized Difference Vegetation Index (NDVI) Harmonics (unitless index)	MODIS (MOD13Q1)
	Enhanced Vegetation Index (EVI) Harmonics (unitless index)	MODIS (MOD13Q1)
	NDVI/EVI Variance and Amplitude (Multi-frequency; unitless index)	MODIS (MOD13Q1)
Hydrological	Monthly Cumulative Precipitation (Jan–Dec; mm)	CHIRPS
	Monthly Relative Humidity (Jan–Dec; %)	ERA5
	Actual Evapotranspiration (ET; mm/day)	GLEAM v3.8
	Open Surface Water Extent (fraction 0–1)	JRC Global Surface Water
	Distance to Permanent Water (km)	HydroSHEDS
Land Cover	Herbaceous Vegetation/Shrubland Cover (fraction 0–1)	ESA WorldCover
	Regularly Flooded Vegetation Cover (fraction 0–1)	ESA WorldCover
	Deciduous Broadleaf/Evergreen Forest Cover (fraction 0–1)	ESA WorldCover
	Mixed and Other Tree Cover (fraction 0–1)	ESA WorldCover
	Cultivated and Managed Vegetation (fraction 0–1)	ESA WorldCover
Ecological	Above-ground Biomass (AGB; Mg/ha)	ESA CCI Biomass
	Distance to Protected Areas (km)	UNEP-WCMC (WDPA)
	Ecological Threat Index (unitless index)	IUCN Red List

All collected predictor datasets were processed, resampled, or aggregated to align spatially with the H3 resolution 7 grid covering India.

### 2.3 Feature extraction and preprocessing

Predictor values were extracted or calculated for each H3 hexagon. Raster predictors were summarized by zonal statistics (mean and standard deviation) using parallelized exactextract. Density variables were log-transformed, and distance metrics were computed in projected coordinate systems appropriate for India. Missing predictor values were imputed with training-fold means within each cross-validation fold. Continuous predictors were standardized within each training fold for models requiring scaling (SVC, KNN, Logistic Regression, and BNN), whereas tree-based models and Gaussian Naive Bayes were trained on the selected predictors without scale-dependent preprocessing.

### 2.4 Model architecture and stacked ensemble

To model the relationships between spatial predictors and avian influenza occurrence, we employed a Stacked Gaussian Process Regressor following Bhatt et al. (15). The first level comprised eight base classifiers: Random Forest, Gradient Boosting, XGBoost, Support Vector Classifier with within-fold StandardScaler, Logistic Regression, K-Nearest Neighbors, Gaussian Naive Bayes, and a Monte Carlo Dropout Bayesian Neural Network. The second level used a Gaussian Process Regressor with a Constant × Matérn(*ν* = 1.5) + WhiteKernel as the meta-learner, trained on out-of-fold predictions from all eight base models. All fold-specific preprocessing steps that estimated parameters from data, including imputation, scaling, and model fitting, were performed within the training portion of each spatial cross-validation fold before evaluation on held-out folds.

The model was trained and validated using 5-fold spatial block cross-validation. The study area was partitioned into 100 blocks on a 10 × 10 grid over projected coordinates (EPSG: 32,644), with blocks randomly assigned to folds to reduce spatial autocorrelation between training and held-out data. Base models were trained on four folds and generated out-of-fold predictions for the held-out fold; this was repeated five times to obtain out-of-fold predictions for all training observations. All eight base models had out-of-fold AUC values above 0.5 (range 0.767–0.851) and were included in the GPR meta-learner. Hyperparameters were optimized using Bayesian optimization guided by the average AUC across spatial cross-validation folds.

Mutual information (MI) feature selection was performed using a k-nearest-neighbor estimator (*k* = 5; scikit-learn mutual_info_classif, random_state = 42) on the balanced training set (1:1 presence-to-absence) prior to cross-validation. The 15 predictors with the highest MI scores were retained for all models. Feature selection was therefore not nested within the primary spatial cross-validation folds; instead, a single global MI-ranked predictor set was defined once from the complete balanced training set and applied unchanged across all folds and in the final national prediction. This fixed-feature approach was used to maintain a stable and interpretable predictor set for the national map and SHAP analysis. Because MI is a supervised step and this global selection was performed once before the primary spatial cross-validation, it could in principle introduce some optimism into the cross-validated performance estimates. To assess the impact of this choice, MI feature selection was additionally repeated within each spatial cross-validation training fold and model performance was compared with the primary fixed-feature analysis. SHAP-based importance for the retained predictors is reported in Supplementary Table S1. Spatial dependence was addressed by assigning spatial blocks as cross-validation groups.

After cross-validation, the SGPR framework was applied to all H3 resolution-7 hexagons across India. Retrained base models generated spatial predictions for the national grid, and these base-model predictions were passed to the GPR meta-learner. The GPR produced two outputs for each hexagon: a relative spatial risk score and a posterior standard deviation used as the prediction-uncertainty estimate.

### 2.5 Gaussian process stacking and uncertainty quantification

**Figure 1:**
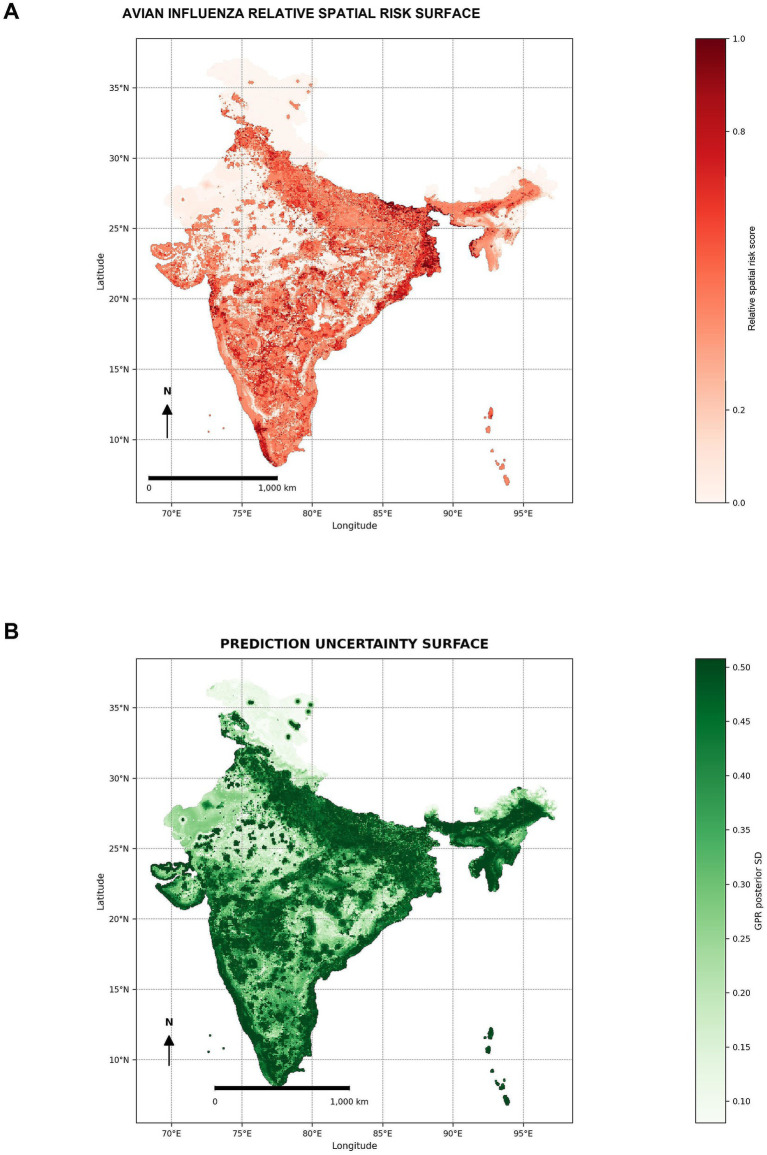
National avian influenza relative spatial risk and prediction uncertainty surfaces. **(A)** Relative spatial risk surface for India at H3 resolution 7 (~5.16 km^2^ per hexagon); warmer colors indicate higher model-estimated relative spatial risk for surveillance prioritization. **(B)** Prediction uncertainty surface, expressed as the GPR posterior standard deviation; darker green areas indicate higher uncertainty and lower model confidence.

Outputs from the selected base models were combined using a Gaussian Process Regressor (GPR) meta-learner trained on out-of-fold predictions. The GPR used a Constant x Matern (nu = 1.5) + WhiteKernel and produced, for each H3 hexagon, a relative spatial risk score and a posterior standard deviation. Throughout this paper, “uncertainty” refers to the GPR posterior standard deviation, which is shown in Figure 1B. To evaluate whether this uncertainty metric was consistent with model disagreement, the GPR posterior standard deviation was compared with the standard deviation of predictions across the selected base models. The two measures were positively correlated (Pearson *r* = 0.74, *p* < 0.001, *n* = 5,000 hexagons), supporting interpretation of the GPR posterior standard deviation as a model-based indicator of prediction confidence for surveillance prioritization. This comparison demonstrates concordance between two model-derived uncertainty summaries and should be interpreted as internal consistency, not as external validation of predictive uncertainty against independent field data.

### 2.6 Model evaluation and diagnostics

Model performance was evaluated from out-of-fold predictions generated under 5-fold spatial block cross-validation. The stacked meta-learner was additionally assessed with a stricter fold-wise spatial holdout in which base learners were fitted on four spatial folds, the GPR was trained from corresponding out-of-fold base-model predictions, and evaluation was performed on held-out hexagons not used for base-learner training. This procedure provided a conservative generalization estimate for the stacked model (AUC = 0.790; 95% CI 0.722–0.854), while the full out-of-fold stacked evaluation produced AUC = 0.853.

Model performance was assessed using the area under the receiver operating characteristic curve (AUC) computed from out-of-fold predictions under 5-fold spatial block cross-validation. Brier score and expected calibration error (ECE, 10 equal-width bins) were computed to assess probabilistic calibration, and reliability diagrams were generated for all base models. Bootstrap 95% confidence intervals for AUC were obtained through 10,000 resamples of the out-of-fold predictions. SHAP (SHapley Additive exPlanations) values were computed using TreeExplainer for the Random Forest model, which had the lowest ECE among individual classifiers, with 100 background samples drawn from the training set. Mean absolute SHAP values were aggregated across all training observations and ranked to produce the quantitative feature-importance Supplementary Table S1.

### 2.7 Spatial surface export and subdistrict summarization

The GPR stacked ensemble produced a relative spatial risk score and a GPR posterior standard deviation for each of the 115,781 H3 resolution-7 hexagons in the prediction grid. These outputs were exported as single-band GeoTIFF rasters in WGS 84 geographic coordinates (EPSG:4326). The spatial risk raster represents the GPR relative spatial risk score on a 0–1 scale, while the uncertainty raster represents the GPR posterior standard deviation. Higher uncertainty values therefore indicate lower model confidence rather than higher disease risk.

Model implementation used PyTorch for the Bayesian neural-network component, and QGIS was used for final cartographic composition (17, 18).

For administrative interpretation, the hexagon-level outputs were summarized to taluk/subdistrict units using the Survey of India subdistrict boundary layer, which provided the most complete national subdistrict geometry available for final mapping. For each subdistrict, the mean GPR relative spatial risk score was stored as mean_risk, and the mean GPR posterior standard deviation was stored as mean_uncertainty. A total of 4,723 Survey of India subdistrict polygons were processed; 4,682 received complete values for both spatial risk and uncertainty, while 41 polygons lacked complete model coverage and were retained as no-data areas.

Two administrative classification products were generated. First, mean_risk was classified into low, medium, and high spatial risk groups to communicate broad administrative gradients in modeled spatial risk. Second, a four-class risk-uncertainty typology was created using median splits among subdistricts with complete values. The median spatial risk threshold was 0.375690, and the median uncertainty threshold was 0.399839. Subdistricts with values greater than or equal to the corresponding median were classified as high spatial risk or high uncertainty; those below the medians were classified as low spatial risk or low uncertainty. Combining these binary indicators produced four classes: low spatial risk + low uncertainty, low spatial risk + high uncertainty, high spatial risk + low uncertainty, and high spatial risk + high uncertainty. The classification was visualized as a single-band categorical GeoTIFF.

## 3 Results

### 3.1 Geospatial dataset for India

**Figure 2:**
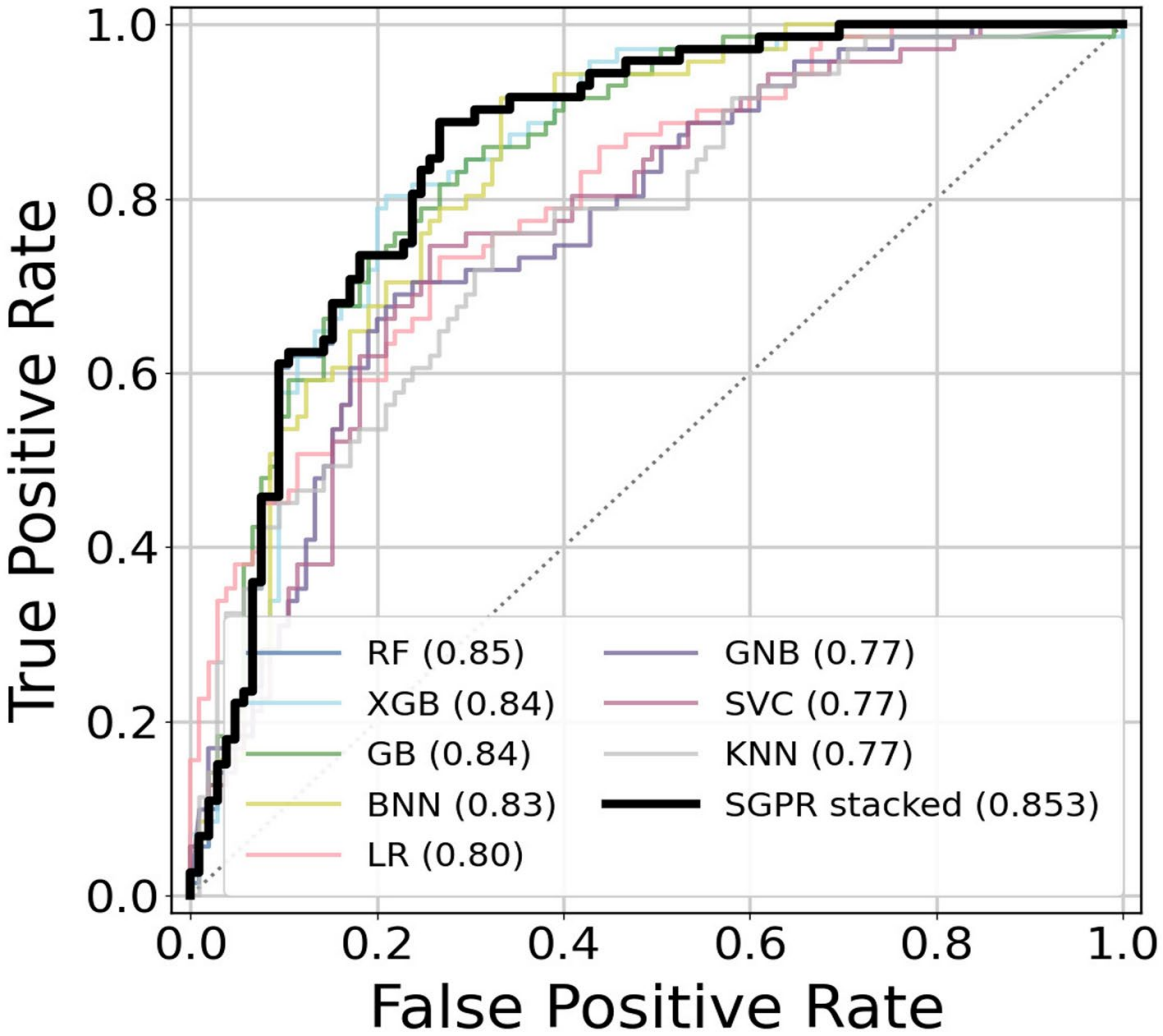
Receiver operating characteristic (ROC) curves for the eight base models and the SGPR stacked meta-learner. The SGPR meta-learner achieved the numerically highest stacked AUC (0.853), while Random Forest was the strongest individual base learner (AUC 0.851); the difference was small and statistically non-significant. The dashed diagonal indicates random classification (AUC = 0.50).

The data integration yielded a national dataset at H3 resolution 7, comprising 71 presence hexagons and 105 buffered pseudo-absence hexagons (176 training hexagons) with 94 extracted features derived from historical H5 AI data, and values for standardized predictor variables covering host, environmental, climatic, and landscape features across India (Figure 2).

The SGPR stacked meta-learner achieved the highest AUC in the final stacked evaluation (AUC 0.853), slightly above Random Forest (AUC 0.851; Brier 0.155; ECE 0.079; bootstrap 95% CI [0.798, 0.907]). The AUC increase over Random Forest was small (Delta AUC = 0.002; bootstrap *p* = 0.30, 10,000 iterations), indicating comparable discrimination between the two models. The SGPR nevertheless provided a posterior uncertainty layer in addition to the relative spatial risk score. Under the stricter spatial holdout, the SGPR achieved AUC 0.790 (95% CI 0.722–0.854). All eight base classifiers performed above chance (AUC > 0.76). In the MI feature-selection sensitivity analysis, fold-specific MI selection produced broadly comparable base-model discrimination to the primary fixed-feature analysis. The mean absolute pooled AUC change across base models was 0.016, and the maximum absolute change was 0.038; fold-specific MI selection retained an average of 10.2 of the 15 predictors from the global top-15 set. The GPR posterior standard deviation was positively correlated with the standard deviation of predictions across the selected base models used in the meta-learner (Pearson *r* = 0.74, *p* < 0.001, *n* = 5,000 hexagons), supporting its interpretation as a model-based indicator of prediction confidence (Table 2).

**Table 2:** Model discrimination and calibration metrics.

Model	AUC	Brier	ECE
SGPR Stacked Ensemble	**0.853**	—	—
Random Forest	0.851	0.155	0.079
XGBoost	0.840	0.178	0.267
Gradient Boosting	0.837	0.201	0.317
BNN (MC Dropout)	0.833	0.171	—
Logistic Regression	0.799	0.182	0.084
SVC (scaled)	0.768	0.195	0.091
Gaussian NB	0.769	0.263	0.307
K-Nearest Neighbors	0.767	0.193	0.199

Bold values indicate the best (highest or lowest as appropriate) performance metric in each column: highest AUC, lowest Brier score, and lowest ECE.

### 3.2 Predictors associated with spatial avian influenza risk

**Figure 3:**
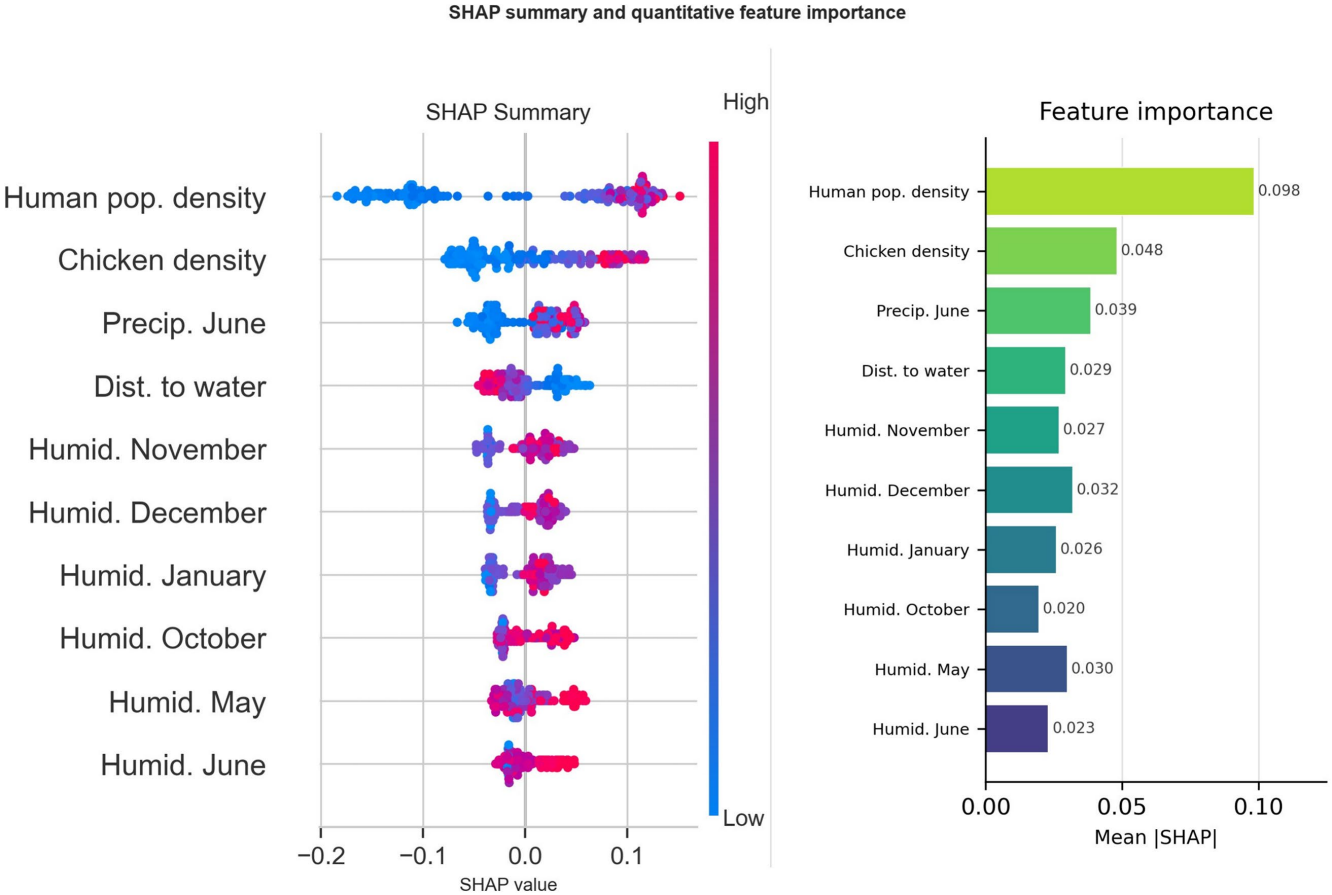
SHAP summary and quantitative feature-importance analysis for the Random Forest model, which had the lowest expected calibration error among individual classifiers (ECE = 0.079). The left panel shows SHAP values for individual training observations colored by feature value, and the right panel shows mean absolute SHAP values. See Supplementary Table S1 for the complete quantitative values.

SHAP analysis ranked human population density (mean |SHAP| = 0.098), extensive chicken density (0.048), June precipitation (0.039), and seasonal humidity variables among the predictors most strongly associated with model predictions. Human population density likely acts as a composite proxy for backyard poultry presence, market activity, and reporting intensity. Multiple poultry density indicators were retained, consistent with the importance of host distribution reported in prior HPAI studies. Because 10 of 15 retained climate predictors had variance inflation factor values exceeding 10, SHAP rankings for monthly humidity and precipitation variables should be interpreted as grouped evidence for seasonal moisture and temperature structure rather than as independent effects of individual monthly variables (Figure 3).

These results indicate that model-estimated spatial avian influenza risk in India was associated with areas characterized by high human and poultry densities, hydrological or wetland-interface conditions represented by distance to water bodies, and seasonal climatic conditions related to temperature variability and moisture.

### 3.3 National spatial avian influenza risk and uncertainty surfaces

The final ensemble produced a national relative spatial risk surface and a companion uncertainty surface for 115,781 H3 resolution-7 hexagons (Figure 1). The spatial risk map (Figure 1A) displays the GPR relative spatial risk score for each hexagon on a 0–1 scale. The uncertainty map (Figure 1B) displays the GPR posterior standard deviation; higher values indicate locations where model predictions warrant greater caution and where additional surveillance may provide higher information value (see Section 2.5 for the uncertainty definition and internal-consistency assessment).

Across 115,781 H3 resolution-7 hexagons, spatial risk had a median of 0.340 (IQR 0.032–0.468; range 0–1) with a top-decile threshold of 0.606. Uncertainty had a median of 0.393 (IQR 0.244–0.491). Higher spatial risk areas were concentrated in the eastern Indo-Gangetic Plain, northeastern India, coastal corridors, and selected southern poultry-producing regions. Lower spatial risk areas were observed in arid western regions, the central highlands, and high-elevation Himalayan grids. Among high spatial risk hexagons, 4,307 also had low uncertainty and represent higher-confidence surveillance targets, whereas 7,273 had high uncertainty and represent data-gap targets for additional field surveillance or predictor refinement.

### 3.4 Subdistrict-level spatial risk and uncertainty classification

**Figure 4:**
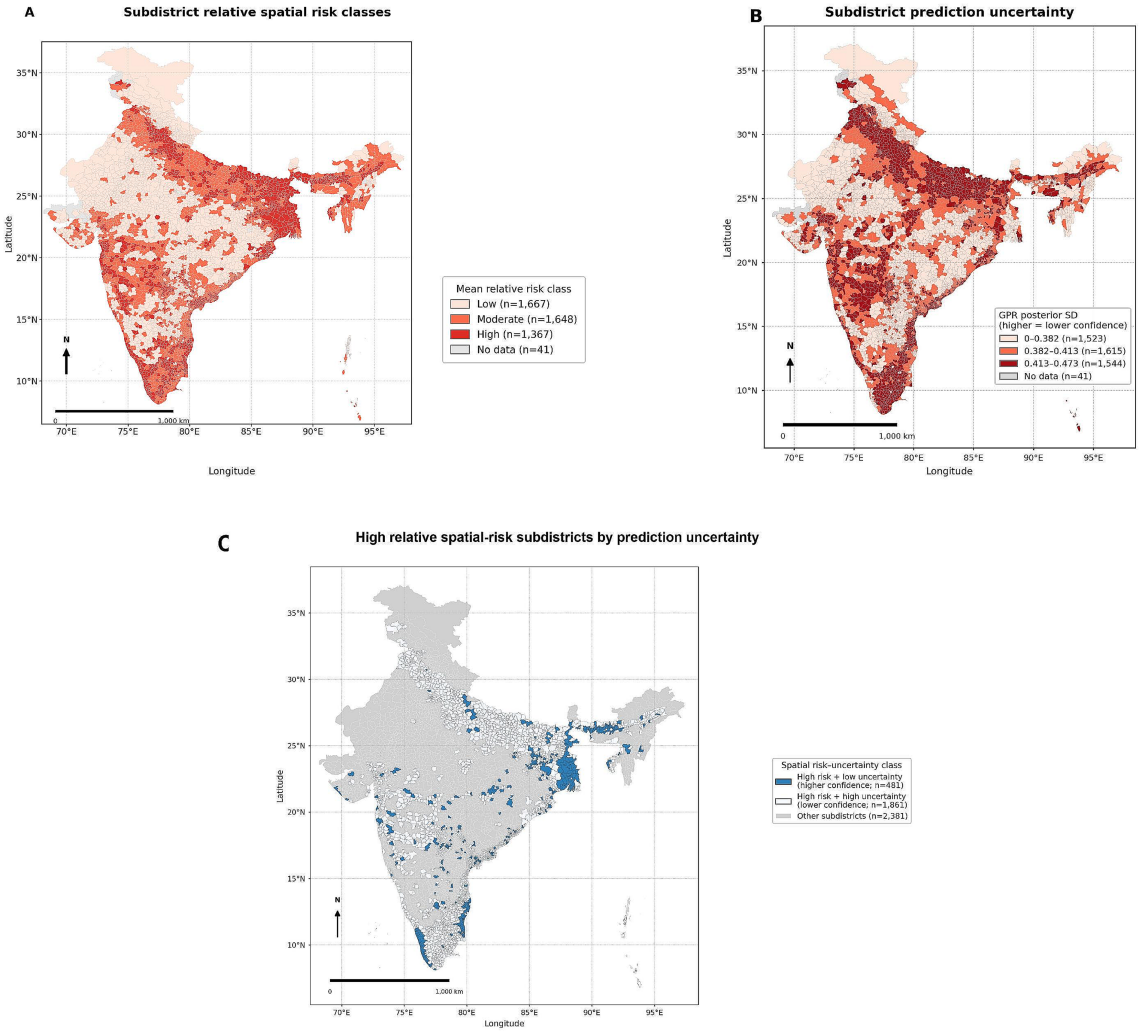
Subdistrict-level relative spatial risk, prediction uncertainty, and high-risk uncertainty classes **(A)** Subdistrict spatial risk classes derived from model-estimated mean relative spatial risk scores summarized to Survey of India subdistrict/taluk boundaries and classified as low, moderate, or high. **(B)** Subdistrict-level prediction uncertainty summarized from the model-derived uncertainty surface; higher values indicate lower model confidence, not higher spatial risk. **(C)** High spatial-risk subdistricts separated by prediction uncertainty using the median uncertainty threshold; blue areas indicate high spatial risk with lower uncertainty and therefore higher model confidence, whereas light areas indicate high spatial risk with higher uncertainty and therefore lower model confidence.

To translate the H3-resolution outputs into an operational administrative framework, predictions were summarized to the complete Survey of India subdistrict/taluk boundary layer. Of 4,723 SOI subdistrict polygons, 4,682 received complete values for both mean spatial risk and mean prediction uncertainty; 41 polygons were retained as no-data areas (Figure 4).

The combined risk-uncertainty classification used median thresholds of 0.375690 for mean spatial risk and 0.399839 for mean uncertainty. It identified 481 subdistricts as high spatial risk + low uncertainty. These areas combine above-median modeled spatial risk with comparatively greater model confidence and represent the strongest surveillance-priority targets in this analysis. A further 1,861 subdistricts were classified as high spatial risk + high uncertainty, indicating elevated modeled spatial risk but lower confidence and therefore a need for both surveillance attention and improved data coverage. The low spatial risk + low uncertainty class contained 1,860 subdistricts, while 480 subdistricts were classified as low spatial risk + high uncertainty.

## 4 Discussion

We developed a national HPAI relative spatial risk mapping framework for India using spatial cross-validation, calibrated model evaluation, and uncertainty mapping. The Gaussian-process stacked meta-learner provided the numerically highest final AUC, while Random Forest was the strongest individual base learner and the difference between the two was small and statistically non-significant. The most directly comparable study, Puttahonnappa et al. (4), applied Random Forest and Support Vector Machine models to HPAI H5N1 outbreaks in India using climatic and environmental predictors, identifying broadly concordant high spatial risk zones in southern and northeastern regions. A formal quantitative spatial concordance analysis between the two studies is precluded by differences in spatial units (administrative districts vs. H3 hexagons at 5.16 km^2^) and the unavailability of per-district spatial risk scores from the earlier study; however, qualitative agreement in the regions identified as high-priority by both approaches supports the robustness of the identified spatial risk patterns. Our framework extends that approach through: (i) H3 hexagonal indexing at 5.16 km^2^ resolution rather than administrative districts, (ii) inclusion of livestock density, land-cover, and anthropogenic predictors alongside climatic variables, (iii) an eight-classifier stacked ensemble with GPR meta-learning, and (iv) formal per-cell uncertainty quantification.

The SGPR framework should be interpreted as an uncertainty-aware extension of the base-learner ensemble rather than as a substantially better discriminator than the best individual classifier. Random Forest remains the strongest individual base model by discrimination and calibration, while the stacked meta-learner provides a companion uncertainty layer that can help distinguish high spatial risk areas with stable model agreement from high spatial risk areas where additional surveillance may have higher expected information value. Thus, the principal added value of the stacked GPR approach in this study is the integration of relative spatial risk scoring with model-based prediction uncertainty. This finding is consistent with Bhatt et al. (15), who showed that the GPR covariance function can capture residual spatial structure after covariate adjustment in disease mapping applications.

The feature-attribution analysis provides context-specific information into the predictor groups associated with model outputs of spatial avian influenza risk within India, based on the historical H5 patterns. The top-ranked predictors, human population density, poultry density, and seasonal climate variables are broadly consistent with those identified by Puttahonnappa et al. (4) for India and by global HPAI risk mapping studies (19–21), suggesting that anthropogenic pressure, host availability, and climatic seasonality are robust cross-study signals despite differences in spatial framework, predictor set, and modeling methodology. The dominance of anthropogenic factors (human population density, urban areas) likely encapsulates a web of factors including backyard poultry density, market networks, trade intensity, and reporting effort. The importance of multiple poultry types (ducks, intensive/extensive chickens) highlights the need for surveillance to encompass India’s diverse production systems. The finding regarding proximity to water bodies requires further ecological study to understand the specific mechanisms operating at these interfaces in India. The role of climate variables, particularly temperature variability and specific seasonal precipitation/humidity patterns, is consistent with prior evidence that climate and hydrological conditions covary with HPAI occurrence.

The national relative spatial risk and uncertainty maps provide operational evidence for surveillance planning. The spatial risk map identifies geographic areas for intensified surveillance, live-bird-market monitoring, focused biosecurity interventions, and rapid response planning. This risk-based approach may support more efficient prioritization compared with uniform strategies, although operational efficiency was not evaluated directly. The uncertainty map complements the spatial risk surface by indicating where predictions are more stable and where high uncertainty warrants cautious interpretation, field investigation, or targeted data collection to improve model confidence.

Several limitations affect interpretation. First, outbreak records derive largely from passive surveillance; modeled relative spatial risk may partly reflect reporting intensity rather than true infection pressure. Second, pseudo-absence locations are sampled background contrasts rather than verified negative locations, so spatial risk scores should be interpreted in relation to the presence-pseudo-absence design rather than as absolute probabilities of occurrence. Third, the analysis uses static or multi-year predictor layers from different time periods (livestock density 2015, human population 2020), while HPAI transmission is temporally dynamic. Fourth, retained climate predictors are highly collinear (10 of 15 with VIF > 10); SHAP values should be interpreted as grouped seasonal-climate signals rather than independent effects of individual monthly variables. Fifth, the primary analysis retained a fixed MI-selected predictor set to support a stable national map and coherent SHAP interpretation. Because MI is a supervised feature-selection step applied globally before the primary spatial cross-validation, this design could in principle introduce some optimism into cross-validated performance estimates. However, fold-specific MI sensitivity analysis showed that this effect was limited: the mean absolute pooled AUC change was 0.016, the maximum absolute change was 0.038, and fold-specific MI selection retained an average of 10.2/15 predictors from the global top-15 set. Fold-specific MI feature-selection sensitivity analysis produced broadly comparable performance, supporting the robustness of the modeling framework; nevertheless, prospective validation against newly reported outbreaks remains important.

Future work should focus on prospective validation of the current spatial risk surface against new outbreak data, incorporation of temporally dynamic predictors including real-time wild bird movement data (e.g., GPS telemetry), poultry trade network information, and extension of the framework to other priority zoonoses.

## 5 Conclusion

We developed and evaluated a stacked machine learning framework for relative spatial avian influenza risk mapping in India at H3 resolution 7. The framework integrates eight base classifiers with a Gaussian-process meta-learner to produce two operational outputs: a national relative spatial risk surface identifying eastern, northeastern, coastal, and selected southern regions as surveillance priorities, and a companion uncertainty layer that showed concordance with ensemble disagreement and distinguishes higher-confidence predictions from areas requiring additional field investigation. Together, the relative spatial risk, uncertainty, and subdistrict classification outputs provide a reproducible basis for risk-based surveillance prioritization under India’s One Health framework.

## Data Availability

The original contributions presented in the study are included in the article/Supplementary material, further inquiries can be directed to the corresponding author.

## Supplementary material

SUPPLEMENTARY TABLE S1 Reports quantitative SHAP feature-importance values for the retained predictors.

SUPPLEMENTARY TABLE S2 Documents the derivation of the final 71 presence hexagons from source outbreak records and literature-derived additions.

SUPPLEMENTARY TABLE S3 Provides subdistrict-level spatial risk and uncertainty summaries, class definitions, data dictionary, and state-level summary counts.

SUPPLEMENTARY FIGURE S1 Shows calibration diagnostics.

SUPPLEMENTARY FIGURE S2 Shows multicollinearity diagnostics based on variance inflation factors.

SUPPLEMENTARY FIGURE S3 Shows model-performance comparison.

SUPPLEMENTARY FIGURE S4 Shows uncertainty diagnostics.

SUPPLEMENTARY FIGURE S5 Shows the Random Forest comparison risk surface.

## References

[1] Bergervoet SA Heutink R Pritz-Verschuren SBE Poen MJ Bouwstra RJ Fouchier RAM . A41 diversity and evolution of avian influenza (AI) viruses in poultry and wild birds. Virus Evol. (2017) 3:vew036.040. doi: 10.1093/ve/vew036.040 28845284 PMC5566070

[2] Galli M Giacomelli A Lai A Zehender G. H5N1 influenza a virus: lessons from past outbreaks and emerging threats. Infez Med. (2025) 33:76–89. doi: 10.53854/liim-3301-7, 40071262 PMC11892436

[3] Bi Y Li J Shi W. The time is now: a call to contain H9N2 avian influenza viruses. Lancet Microbe. (2022) 3:e804–5. doi: 10.1016/S2666-5247(22)00232-4, 36115378

[4] Puttahonnappa SK Anandakumar J Barman NN Rajkumar R Paramanandham K Patil SS . Investigating environmental determinants and spatiotemporal dynamics of highly pathogenic avian influenza H5N1 outbreaks in India through machine learning. Sci Rep. (2025) 15:36132. doi: 10.1038/s41598-025-96765-2, 41102300 PMC12533099

[5] Mishra AC Cherian SS Chakrabarti AK Pawar SD Jadhav SM Pal B . A unique influenza a (H5N1) virus causing a focal poultry outbreak in 2007 in Manipur, India. Virol J. (2009) 6:26. doi: 10.1186/1743-422X-6-26, 19236725 PMC2654876

[6] Dhingra MS Dissanayake R Negi AB Oberoi M Castellan D Thrusfield M . Spatio-temporal epidemiology of highly pathogenic avian influenza (subtype H5N1) in poultry in eastern India. Spat Spatiotemporal Epidemiol. (2014) 11:45–57. doi: 10.1016/j.sste.2014.06.003, 25457596

[7] Pandit PS Bunn DA Pande SA Aly SS. Modeling highly pathogenic avian influenza transmission in wild birds and poultry in West Bengal, India. Sci Rep. (2013) 3:2175. doi: 10.1038/srep02175, 23846233 PMC3807259

[8] Fuller TL Saatchi SS Curd EE Toffelmier E Thomassen HA Buermann W . Mapping the risk of avian influenza in wild birds in the US. BMC Infect Dis. (2010) 10:187. doi: 10.1186/1471-2334-10-187, 20573228 PMC2912310

[9] Musa E Nia ZM Bragazzi NL Leung D Lee N Kong JD. Avian influenza: lessons from past outbreaks and an inventory of data sources, mathematical and AI models, and early warning Systems for Forecasting and Hotspot Detection to tackle ongoing outbreaks. Healthcare (Basel). (2024) 12:1959. doi: 10.3390/healthcare12191959, 39408139 PMC11476403

[10] Artois J Vergne T Fourtune L Dellicour S Scoizec A Le Bouquin S . Spatial risk modelling of highly pathogenic avian influenza in France: fattening duck farm activity matters. PLoS One. (2025) 20:e0316248. doi: 10.1371/journal.pone.0316248, 39903711 PMC11793745

[11] Gilbert M Pfeiffer DU. Risk factor modelling of the spatio-temporal patterns of highly pathogenic avian influenza (HPAIV) H5N1: a review. Spat Spatiotemporal Epidemiol. (2012) 3:173–83. doi: 10.1016/j.sste.2012.01.002, 22749203 PMC3389348

[12] East IJ Hamilton S Garner G. Identifying areas of Australia at risk of H5N1 avian influenza infection from exposure to migratory birds: a spatial analysis. Geospat Health. (2008) 2:203–13. doi: 10.4081/gh.2008.244, 18686269

[13] Mayfield HJ Smith CS Lowry JH Watson CH Baker MG Kama M . Predictive risk mapping of an environmentally-driven infectious disease using spatial Bayesian networks: a case study of leptospirosis in Fiji. PLoS Negl Trop Dis. (2018) 12:e0006857. doi: 10.1371/journal.pntd.0006857, 30307936 PMC6198991

[14] Akter R Hu W Gatton M Bambrick H Cheng J Tong S. Climate variability, socio-ecological factors and dengue transmission in tropical Queensland, Australia: a Bayesian spatial analysis. Environ Res. (2021) 195:110285. doi: 10.1016/j.envres.2020.110285, 33027631

[15] Bhatt S Cameron E Flaxman SR Weiss DJ Smith DL Gething PW. Improved prediction accuracy for disease risk mapping using gaussian process stacked generalization. J R Soc Interface. (2017) 14:20170520. doi: 10.1098/rsif.2017.0520, 28931634 PMC5636278

[16] Walsh MG Mor SM Hossain S. Highly pathogenic avian influenza (H5N1) landscape suitability varies by wetland habitats and the degree of Interface between wild waterfowl and poultry in India. Viruses. (2020) 12:1290. 33187179 10.3390/v12111290 PMC7697759

[17] Paszke A Gross S Massa F Lerer A Bradbury J Chanan G . "PyTorch: an imperative style, high-performance deep learning library". In: Advances in Neural Information Processing Systems. Red Hook, NY: Curran Associates, Inc. (2019). p. 32.

[18] QGIS Development Team QGIS Geographic Information System (2024). Beaverton, OR: Open Source Geospatial Foundation (OSGeo). Available online at: https://qgis.org (Accessed March 15, 2026).

[19] Li Y Qiao Y Zhan Y Dong J van Toor M Waldenström J . Mapping global avian influenza risk patterns through waterbird activity entropy. Nat Commun. (2026) 17:3606. doi: 10.1038/s41467-026-70432-0, 41794845 PMC13096158

[20] Jindal M Lim S MacIntyre CR. Machine learning-based geospatial risk modeling of global avian influenza outbreaks. Transbound Emerg Dis. (2026) 2026:6615342. doi: 10.1155/tbed/6615342 42022449 PMC13095849

[21] Dupas MC Vincenti-Gonzalez MF Dhingra M Guinat C Vergne T Wint W . Global risk mapping of highly pathogenic avian influenza H5N1 and H5Nx in the light of epidemic episodes occurring from 2020 onwards. eLife. (2026) 14:RP104748. doi: 10.7554/eLife.104748, 41603362 PMC12851579

